# Biodegradation and Detoxification of Azo Dyes by Halophilic/Halotolerant Microflora Isolated From the Salt Fields of Tibet Autonomous Region China

**DOI:** 10.3389/fmicb.2022.877151

**Published:** 2022-05-10

**Authors:** Hulin Qiu, Fengfei Shen, Aiguo Yin, Jiaxian Liu, Biyu Wu, Ying Li, Yunyi Xiao, Jinping Hai, Bo Xu

**Affiliations:** ^1^College of Biological and Food Engineering, Guangdong University of Petrochemical Technology, Maoming, China; ^2^Guangdong Laboratory for Lingnan Modern Agriculture, Maoming, China

**Keywords:** azo dyes, biodegradation, decolorization, halophilic/halotolerant microflora, detoxification

## Abstract

This study aimed to decolorize azo dyes in high-salt industrial wastewater under high-salt and low oxygen conditions using extreme halophilic/halotolerant bacteria screened from the salt fields of Tibet, which consisted of *Enterococcus*, unclassified *Enterobacteriaceae*, *Staphylococcus*, *Bacillus*, and *Kosakonia*. Under the optimal conditions, 600 mg/l Congo red, Direct Black G (DBG), Amaranth, methyl red, and methyl orange could be completely decolorized in 24, 8, 8, 12, and 12 h, respectively. When the DBG concentration was 600 mg/l, NADH–DCIP, laccase, and azo reductase were confirmed to be the primary reductase and oxidase during the degradation process, and the degradation pathways were verified. The microflora could not only tolerate changes in salt concentrations of 0–80 g/l, but also displayed strong degradative ability. Under high-salt concentrations (≥ 60 g/l NaCl), NADH–DCIP reductase was primarily used to decolorize the azo dye. However, under low salt concentrations (≤ 40 g/l NaCl), azo reductase began to function, and manganese peroxidase and lignin peroxidase could cooperate to participate in DBG degradation. Additionally, the halophilic/halophilic microflora was shown to convert the toxic DBG dye to metabolites of low toxicity based on phytotoxicity analysis, and a new mechanism for the microflora to degrade DBG was proposed based on intermediates identified by liquid chromatography-mass spectrometry (LC–MS). This study revealed that the halophilic/halophilic microflora has effective ecological and industrial value for treating wastewater from the textile industry.

## Introduction

Dyes are widely used in the food, textile, plastic, leather, and printing industries. However, the complexity, toxicity, and stability of dyes have increased in parallel with the industrial demand for dye color and fastness, which simultaneously results in more serious environmental pollution. There are more than 1,000 kinds of dyes used in industries that have a global output of 280,000 tons every year, and azo dyes comprised more than 60% of the synthetic dyes (Patel et al., 2017; Pattanaik et al., 2020). Statistically, 30–50% of these dyes are directly discharged into natural water systems without treatment (Giovanella et al., 2020), causing a substantial amount of serious water pollution near the printing and dyeing industries. The degradation of these dyes results in toxic aromatic amines that are highly persistent and have numerous adverse effects on humans and aquatic organisms (Kurade et al., 2015). Owing to the stable and strong coloring power of azo dyes, their chemical structure contains one or more azo bonds. Thus, they can resist degradation by most microorganisms. The concentration of residual azo dyes in the wastewater of dye industries is approximately 10–200 mg/l. Direct discharge can not only be esthetically unpleasant but also result in catastrophic consequences for aquatic organisms owing to their chemical toxicity. Currently, the treatment methods for azo dyes primarily include physical adsorption, membrane filtration, photocatalysis, coagulation, and ozone oxidation (Lucas et al., 2006; Dafale et al., 2008; Khan et al., 2014; Dutta et al., 2015). However, the by-products of azo dye processing are highly toxic, costly, time-consuming, and labor-intensive; have a low efficiency; and are unsuitable for labor-intensive industries, which are the primary disadvantages of these traditional technologies (Chen et al., 2015; Martorell et al., 2017). As an effective treatment for azo dye wastewater, bioremediation has received increasing amounts of attention in recent years because this biological method of treating printing and dyeing wastewater has many advantages, including that it is eco-friendly, has low disposal costs, generates less secondary waste, and can mineralize azo dye molecules (Oturkar et al., 2011; Naresh et al., 2013; Alegbeleye et al., 2017).

In the past few years, researchers have discovered that many bacteria, fungi, algae, and plants can degrade azo dyes (Iqbal et al., 2011; Agrawal et al., 2014; Zhang et al., 2015; Ali et al. 2019; Giovanella et al., 2020). Undoubtedly, bacteria have the highest potential and are the most promising because they have many advantages, such as a short life cycle and they quickly degrade compounds into metabolites that are much less toxic and gentle to the environment. Simultaneously, bacteria can also grow on the surface of various substrates without environmental restrictions (Kolekar et al., 2012). Typically, the degradation process of azo dyes by microorganisms is a continual aerobic–anaerobic process. The decolorizing of azo dyes, which produce various aromatic hydrocarbons and aromatic amines, can be completed during the anaerobic process, while the aerobic process is a detoxification process and helps to mineralize the aromatic hydrocarbons and aromatic amines. However, since most azo dyes are difficult to degrade under aerobic conditions, their applications are limited. In recent years, research on the biodegradation of azo dyes under microaerobic conditions has been relatively limited (Khan et al., 2014; Lade et al., 2015). The rate of biodegradation of azo dyes primarily depends on the type of bacteria utilized for this purpose. Compared with purified single bacteria species, a mixture of bacteria would be more effective for the bacterial metabolism and degradation of azo wastewater (Kolekar et al., 2012) because a quicker degradation efficiency would be gained through the synergistic action of microorganisms and enzymes in the presence of mixed bacteria (Fang et al., 2013). Therefore, it is critical to develop a rich and diverse microbial community to effectively degrade azo dyes. Typically, industrial textile wastewater contains azo dyes and high concentrations of salt, which induce higher osmotic pressure (Khalid et al., 2012; Liu et al., 2013; Guo et al., 2020). This limits the types of microorganisms that can degrade textile wastewater. However, the halotolerant and halophilic microorganisms have specific natural advantages for coping with high osmotic pressure during the treatment of azo wastewater, particularly for high-salt azo dye wastewater. Halotolerant and halophilic microorganisms are unique groups of organisms that can live in high salinity environments. As there are high (3–10%) concentrations of NaCl contained in dyeing wastewater (Giovanella et al., 2020; Guo et al., 2020), during the process of treating and degrading textile wastewater, it would be ideal to obtain microbial strains that could tolerate such high-salt concentrations. Because halotolerant/halophilic microorganisms can survive in an excessively high-salt environment, they would be the best option to decolorize and degrade azo dyes with a high-salt content in wastewater.

The bioremediation of extreme environments requires microbial populations that are adapted to the environment. Many bacteria and filamentous fungi with the ability to degrade azo dyes have been reported. However, current research about the degradation by halotolerant biological communities is relatively limited and primarily focused on the degradation of azo dyes by halotolerant single-cell fungi, such as yeast (Song et al., 2017; Al-Tohamy et al., 2020; Guo et al., 2020). Some strains with the ability to tolerate various extreme conditions, such as high salt, include *Pichia pastoris*, *Streptomyces halophilus*, and *Scheffersomyces spartinae*, among others that have been identified recently (Song et al., 2017; Sameh Ali et al., 2020). However, to date, there have been no reports on the decolorization and degradation of azo dyes by microorganisms isolated from halophilic/halotolerant biomes. Compared with degradation by single bacteria species, composite colonies are more effective at degrading pollutants because of the interactions among different bacteria and their enzymes.

In this study, a halophilic/halotolerant microbial population that was able to degrade azo dyes under micro-oxygen conditions was successfully isolated from a salt mine in Yanjing Town, Mangkang County, Tibet Autonomous Region, China (29°04.814′N, 98°59.405′E). Owing to the high salt and osmotic pressure of the azo wastewater, treatment of the dye wastewater by the halophilic/halotolerant microorganisms was considered to be a practical and reliable method. Therefore, based on the successful isolation of the microflora, this study further examined the decolorization and degradation process of azo dyes of various structures by the bacterial flora, and the subsequent experiments were primarily conducted with DBG (CAS number 6428–31–5) in the latter period. Simultaneously, the impact of key parameters, such as pH, salinity, dye concentration, and different dye structures of azo dyes, on microbial degradation was also studied in this experiment. Moreover, the diversity of halophilic/halotolerant microflora was determined *via* high-throughput sequencing analysis, and the various intermediates were identified using GC–MS. Finally, a possible DBG degradative pathway by the microflora was proposed in this study. These results are expected to provide an effective biodegradation strategy to treat dyeing wastewater that contains azo dyes and high salt.

## Materials and Methods

### Dyes and Chemicals

Direct Black G (DBG; CAS number 6428–31–5) is a commonly used azo dye. Owing to its high content, high relative molecular weight, complex structure, and multiple stable azo bonds, it is difficult to degrade in the environment. Therefore, in this experiment, DBG was used as the main degradation dye. Other azo dyes with different structures, including Direct Black 38, Congo red, Amaranth, and methyl orange, were chosen to examine the decolorizing capability of the isolated microflora. The structures and characteristic absorption wavelengths of the five azo dyes used in this study are presented in Table 1. All the chemicals used were of analytical grade.

**Table 1:** Characteristics of the main azo dyes used in this study.

Azo dyes	Molecular structures	Wavelength (nm)
Direct Black G	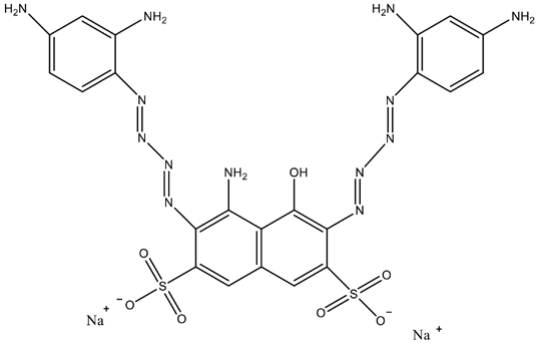	645
Direct Black 38	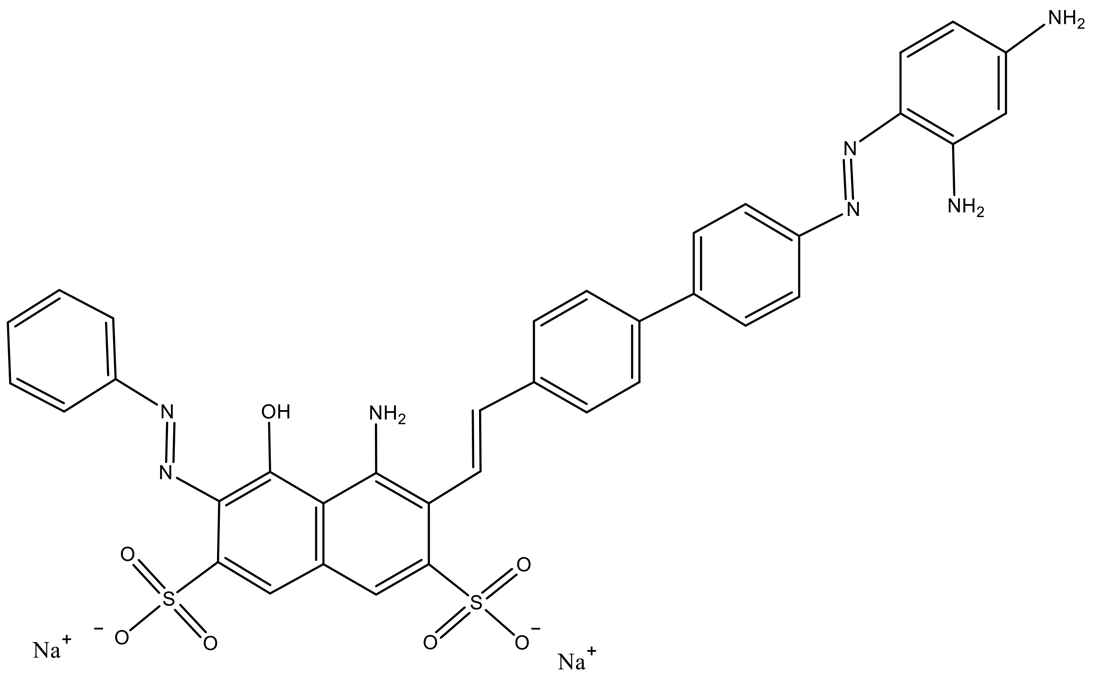	590
Congo Red	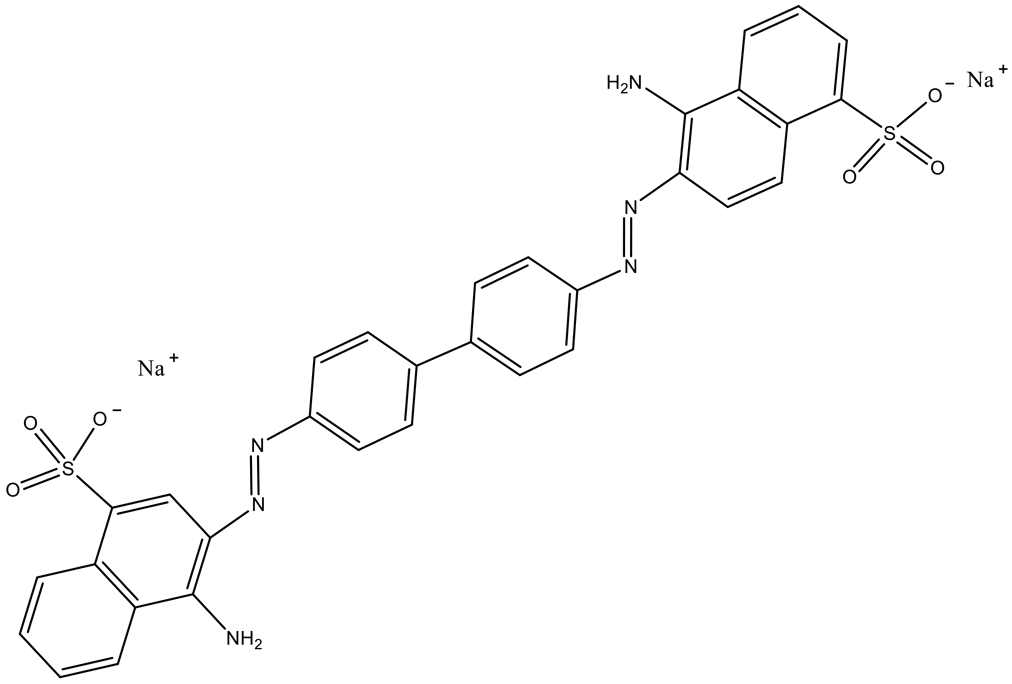	493
Methyl Orange	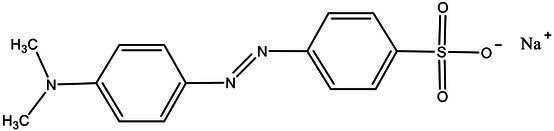	465
Amaranth	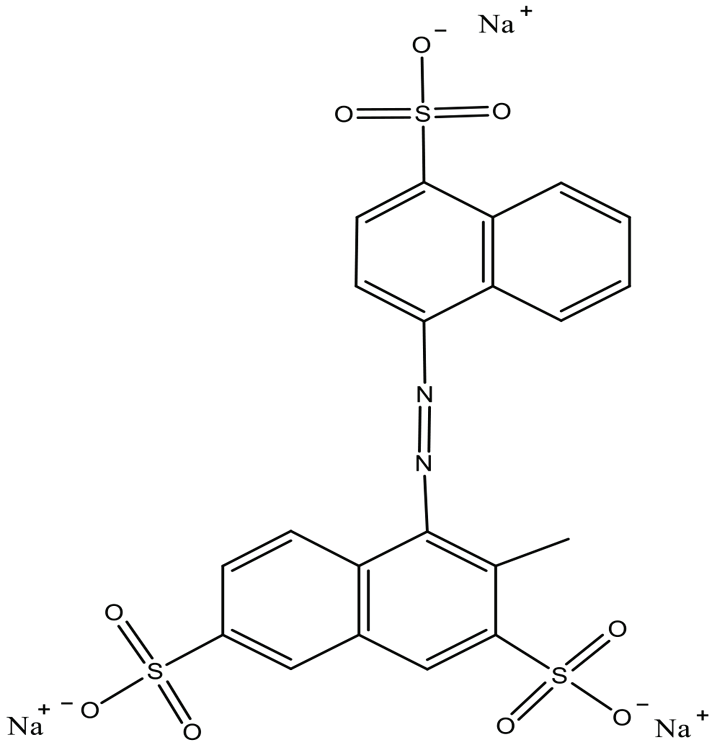	522
Methyl red	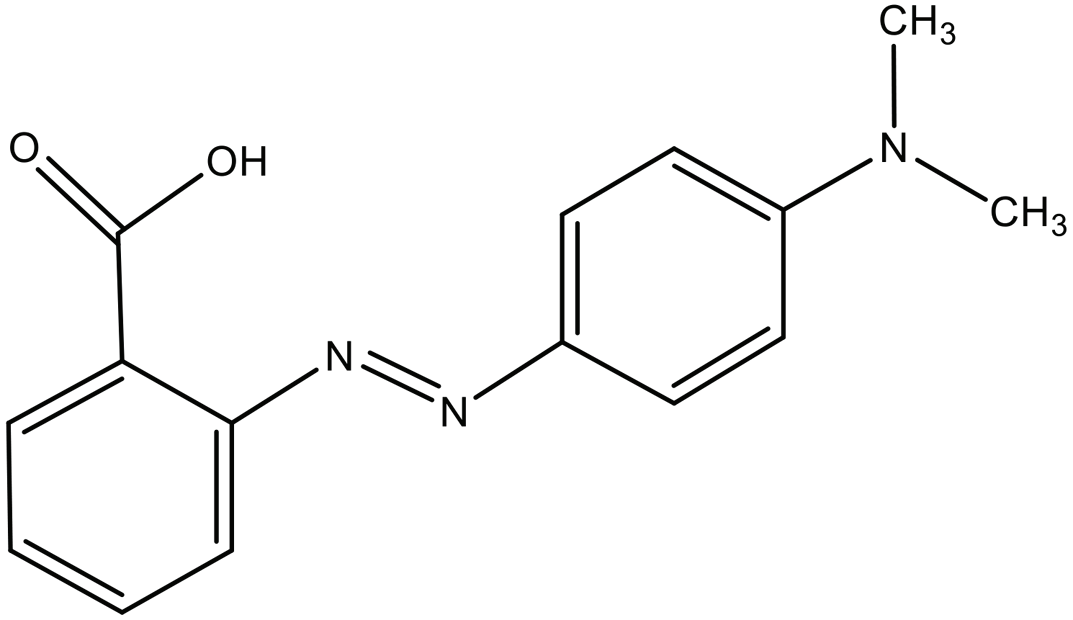	515

### Media

TSB was used as enrichment of the media in this study. Degradation media that contained 5.0 g of glucose, 2.0 g of peptone, 2.0 g of soy protein, 1.80 g of KH_2_PO_4_, and 3.50 g of NaH_2_PO_3_ per liter of distilled water was used, and the pH was adjusted to 7.0 with 2 M NaOH. The rest of the preparation included adding different concentrations of azo dyes to the degradation media (i.e., DBG, Direct Black 38, Amaranth, Congo red, and methyl orange).

### Development of Halophilic/Halotolerant Microflora Capable of Decolorizing Azo Dyes

The microflora was screened for isolates that could decolorize azo dyes. The microflora originated from the Millennium Ancient Salt Village in Yanjing Town, Mangkang County, Tibet Autonomous Region, China (29°04.814′N, 98°59.405′E). The crude salt samples were stored at 4°C for transportation to the laboratory. Forty grams of crude salt were dissolved in TSB (tryptone, 17.0 g; sodium chloride, 5.0 g; soy peptone, 3.0 g; glucose, 2.5 g; and dipotassium phosphate, 2.5 g; pH 7.3) and cultured at 37°C for 3 days for enrichment. A volume of 5 ml of enriched bacteria was placed in a 250 ml Erlenmeyer flask that contained 100 mg/l of dye. The enrichment culture in the inoculating flask was grown under microaerobic conditions at 37°C. After 3 days, 5 ml of culture medium was transferred to 100 ml of fresh degradation medium to continue culturing and acclimatize it to completely decolorize under the same conditions. When the decolorization became stable, the concentration of the dye was increased to 200, 300, 400, 500, and 600 mg/l, respectively. The final stable decolorization of 600 mg/l dye represented the successful decolorization by the microflora.

### Decolorization Studies

All the decolorization experiments were conducted in Erlenmeyer flasks. The halophilic/halotolerant microbial communities were pre-cultured in decolorizing medium that contained 600 mg/l DBG. Subsequently, various factors that affect the determination were adjusted, including the dye concentration (200–3,600 mg/l), salt concentration (0–160 mg/l), and temperature (30–55°C). With a shaker speed of 0–200 rpm, the decolorization ability of dyes with different structures (i.e., DBG, Direct Black 38, Congo red, Amaranth, and methyl orange) was determined according to the following equation:


$$\mathrm{Decolorization}\;\left(\%\right)=\frac{A0-A1}{A0}\times 100\%\text{.}$$

### Microbial Diversity Analysis

Microbial genomic DNA was extracted using an E.Z.N.A.^®^ Soil DNA Kit (Omega Bio-tek, Norcross, GA, United States) according to the manufacturer’s instructions. The DNA extract was checked on a 1.0% agarose gel, and the DNA concentration and purity were determined with a NanoDrop 2000 UV–Vis spectrophotometer (Thermo Scientific, Waltham, MA, United States). The *V3–V4* hypervariable region of the bacterial 16S rRNA gene was amplified with the primer pair 338F (5′-ACTCCTACGGGAGGCAGCAG-3′) and 806R (5′-GGACTACHVGGGTWTCTAAT-3′) on an ABI GeneAmp 9,700 PCR thermocycler (Applied Biosystems, Waltham, MA, United States). PCR amplification of the 16S rRNA gene was performed as follows: initial denaturation at 95°C for 3 min; followed by 27 cycles of denaturation at 95°C for 30 s, annealing at 55°C for 30 s, and extension at 72°C for 45 s; and with a single extension at 72°C for 10 min and a final holding temperature of 4°C. The PCR mixtures contained 4 μl of 5 × *TransStart* Fast Pfu buffer, 2 μl of 2.5 mM dNTPs, 0.8 μl of forward primer (5 μM), 0.8 μl of reverse primer (5 μM), 0.4 μl of *TransStart* FastPfu DNA Polymerase, and 10 ng of template DNA, with ddH_2_O added to reach a final reaction volume of 20 μl. PCR amplifications were performed in triplicate. The PCR product was extracted from 2.0% agarose gel and purified using an AxyPrep DNA Gel Extraction Kit (Axygen Biosciences, Union City, CA, United States) according to manufacturer’s instructions and quantified using a Quantus^™^ Fluorometer (Promega, Madison, WI, United States). Purified amplicons were pooled in equimolar amounts and paired-end sequenced on an Illumina MiSeq PE300 platform/NovaSeq PE250 platform (Illumina, San Diego, CA, United States) according to the standard protocols of Majorbio Bio-Pharm Technology Co. Ltd. (Shanghai, China). The raw reads were deposited into the NCBI Sequence Read Archive (SRA) database (Accession Numbers SAMN23288608–SAMN23288610).

### Enzymatic Assays

The halophilic/halotolerant microflora were incubated in degradation media that contained 600 mg/l of DBG and 60 g/l NaCl at 20°C for 24 h (pH 8.0) and harvested by centrifugation at 8,000 × *g* for 15 min. All the enzymes that were extracted from the cells and supernatant were assayed using a UV–Vis spectrophotometer. The absorbance of the supernatant before and after degradation was measured by full wavelength scanning. The collected cells were washed in PBS three times and treated with lysozyme for 20 min at 50 rpm, with 10 strokes of 20 s each, separated by 10 s intervals for 15 min at 4°C. The processed homogenate was again centrifuged, and the supernatant was used as the source of crude enzymes. Similar procedures were followed to extract enzymes from the control medium. The activities of laccase, lignin peroxidase, and manganese peroxidase, azo reductase, and NADH–DCIP reductase were monitored spectrophotometrically in cell-free extracts, as well as in the control supernatant using a UV–Vis spectrophotometer.

Laccase activity was determined by monitoring the increase in optical density at 420 nm (420 nm = 36,000 [mol/L]^−1^ cm^−1^) in a 4-ml reaction mixture that contained 1 ml of 1 mM diammonium 2,2′-azino-bis[3-ethylbenzothiazoline-6-sulfonate (ABTS)] in 100 mM acetate buffer (pH 4.0; Wolfenden and Willson, 1982). Lignin peroxidase activity was estimated by monitoring the formation of veratraldehyde at 310 nm (310 nm = 93,000 [mol/L]^−1^ cm^−1^) in a 3-mL reaction mixture that contained 10 mM resveratrol, 10 mM H_2_O_2_, and 100 mM tartaric acid (pH 3.0; Kalyani et al., 2008). Manganese peroxidase activity was assayed at 240 nm (240 nm = 6,500 [mol/L]^−1^ cm^−1^), and the 3-mL reaction mixture contained 10 mM H_2_O_2_ and 15 mM MnSO_4_ in 50 mM succinate buffer (pH 4.5). Azo reductase activity was assayed as described by Wolfenden and Willson (1982). NADH–DCIP reductase activity was assayed at 590 nm as described by Saratale et al. (2009). All the enzyme assays were conducted at 30°C and the reference blanks contained all the components except for the enzyme. All the enzymes were assayed in triplicate, and the average rates were calculated. One unit of enzyme activity was defined as a change in absorbance units of min^−1^ mg of protein^−1^. The protein content was determined as described by Lowry et al. (1951) with bovine serum albumin used as the standard.

### LC–MS Analyses

The metabolites after decolorization of DBG (600 mg/l) by the microflora were extracted. Briefly, 100-mL decolorized broth samples were collected and centrifuged at 10,000× *g* for 10 min to obtain the cell-free supernatant. An equal volume of ethyl acetate was added to the supernatant three times to extract the intermediate products. The extract was obtained after evaporation to dryness under vacuum at 45°C with a rotary evaporator (NE-52AA, Yarong Biochemical Instrument Co., Ltd., Shanghai, China) and dissolved with methanol. The solution was filtered through a 0.22-μm filter membrane before analysis.

### Toxicity Studies

Assessment of any bioremediation technology needs to evaluate the toxicity of the original dyes and their metabolites after decolorization, and the toxicity of degradation products is more important than the decolorization process itself. Thus, acute toxicity assessment of DBG and its products was obtained after degradation by halophilic/halotolerant flora. The toxicity test of DBG and its produced metabolites was performed on three types of seeds commonly used in agriculture: corn (*Zea mays*), rice (*Oryzae sativa*), and wheat (*Triticum aestivum*). The DBG powder was dissolved in distilled water to a final concentration of 600 mg/l, and degraded supernatant was passed through a 0.22-μm filter after removing salt with a dialysis bag (3,500 D). The toxicity test was conducted by watering the seeds of each plant with 10 ml of DBG solution and its degradation metabolites. The control was maintained by irrigating the seeds in 10 ml of distilled water. After culture in a 25°C incubator for 4 days, the germination rate (%) per 100 seeds was recorded, and the length of the shoots and roots was recorded after 8 days. The following equation was used to calculate the percentage of germination:


$$\mathrm{Germination}\;\left(\%\right)=\frac{\mathrm{No}.\mathrm{of seeds germinated}}{\mathrm{No}.\mathrm{of seeds sowed}\;10}\times 100.$$

## Statistical Analysis

All data other than those associated with optimization were statistically analyzed using one-way analysis of variance (ANOVA) and a Tukey–Kramer multiple comparison test.

## Results and Discussion

### Microbial Diversity Analysis

**Figure 1:**
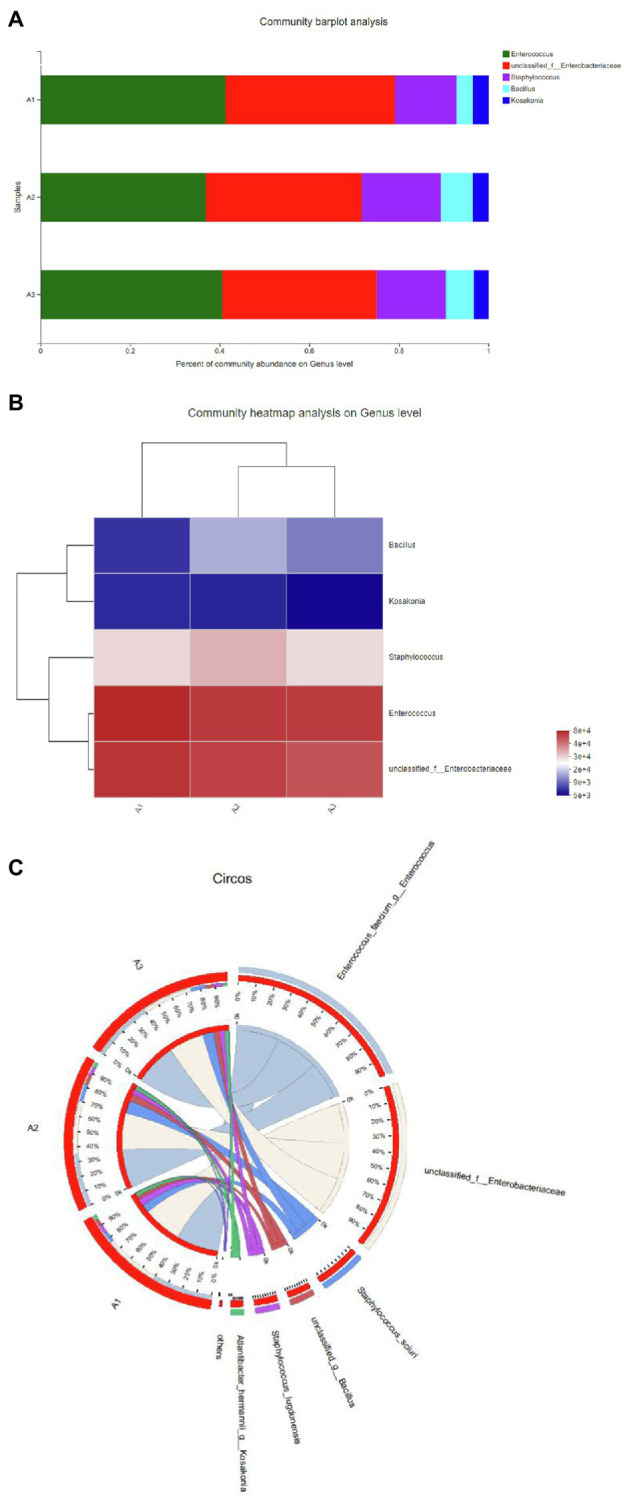
**(A)** The distribution of each sample at the genus level. **(B)** Sample and genus relationship. **(C)** The abundance changes of different bacteria in each sample at the genus level. The right side of the figure shows the value represented by the color gradient.

In this study, bacteria were collected from decolorized media, and DNA was extracted from the cells for high-throughput sequencing. After testing in three parallel groups, the extreme halophilic/halotolerant flora was determined to mainly consist of five genera of bacteria. Bacteria grew well in the media with high osmotic pressure and a high concentration of azo dyes and could participate in the biotransformation process. Five genera were detected in three samples (Figure 1A). The most abundant were *Enterococcus* within Firmicutes and *Enterobacteriaceae* within Proteobacteria. A total of 75% of the bacteria in the flora were composed of *Enterococcus* and *Enterobacter*, and the rest were *Staphylococcus*, *Cosaconia*, and *Bacillus* (Figure 1B). *Enterococcus* and *Enterobacter* were the most adaptable to high-salt environments (Figure 1C). Previous studies have shown that *Enterobacter* and *Enterococcus* can degrade azo dyes of different structures (Table 2) because they can produce azo reductase (Chalansonnet et al., 2017; Eslami et al., 2019; Zahran et al., 2019). Some types of *Bacillus* are thermophilic. Their presence enabled the flora to degrade dyes at 50°C. In addition, there were also reports that different types of azo dyes could be degraded by strains of *Bacillus, Brevibacterium*, and *Geobacillus* (Abbas et al., 2020; Romero et al., 2020; Krithika et al., 2021). These strains can secrete azo reductase and laccase during the degradation process and could degrade azo dyes together. Among these bacteria, new types of *Enterococcus* and *Bacillus* have been found. *Staphylococcus ludunensis* (Zipperer et al., 2016) has also attracted attention because of its metabolites with broad-spectrum antibacterial activity. *Enterococcus* and *Enterobacter* can grow in a high-salt environment and are strongly resistant to such an environment, so they can work in concert to create conditions for the degradation of azo dyes by consuming the toxic substances produced by the degradation of these dyes.

**Table 2:** The ability of extreme halophilic/halotolerant bacteria to degrade azo dyes with different structures.

Azo dye	Molecular structure	Liquid medium		600 mg/l (24 h) Maximum decolorization (%)
		Before decolorization	After decolorization	
Congo red	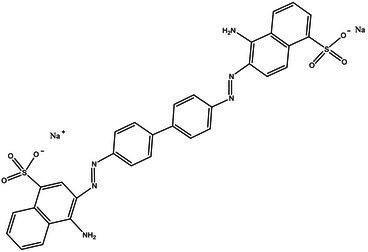	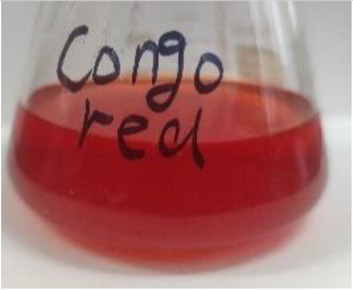	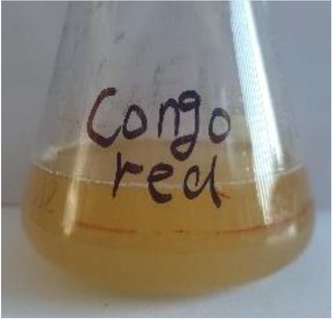	93.3
Methyl orange	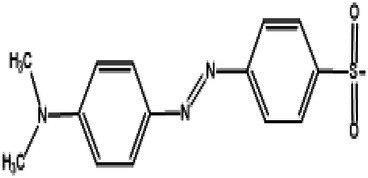	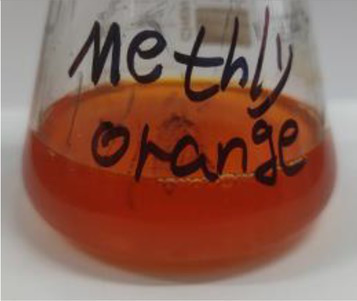	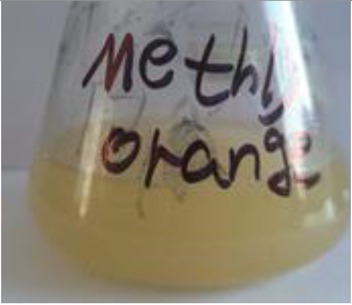	98.6
Direct Black G (DBG)	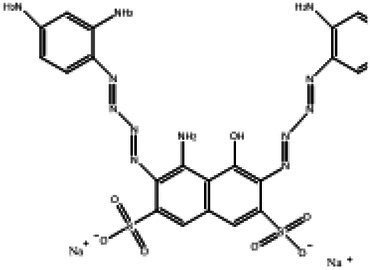	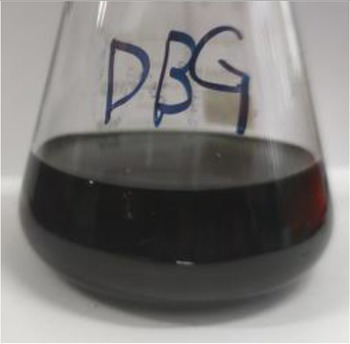	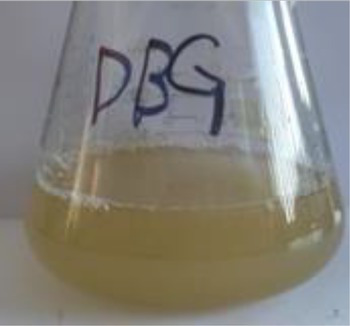	96.1
Methyl red	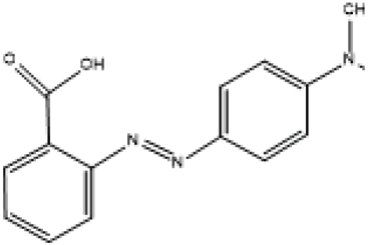	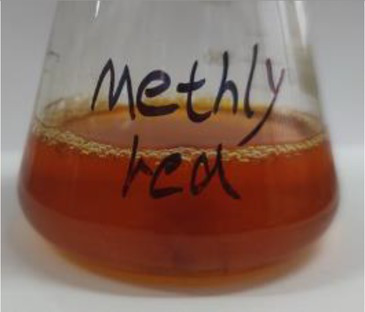	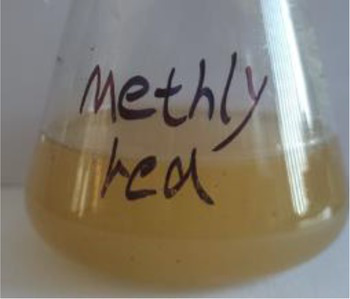	98.4
Amaranth	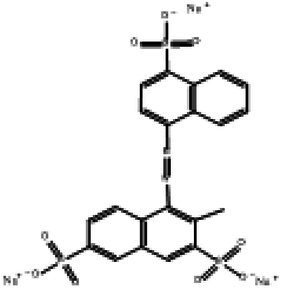	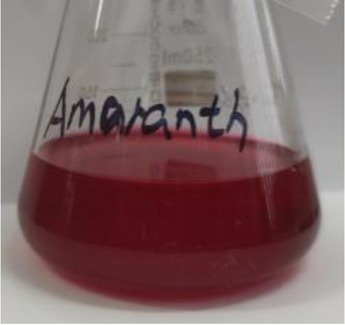	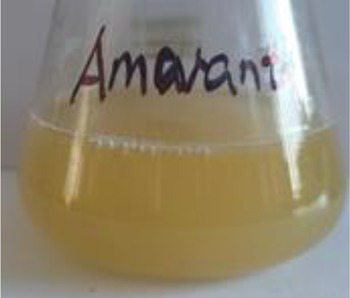	99.2

### Effects of Different Parameters on DBG Decolorization

#### The Effect of Temperature on Decolorization

**Figure 2:**
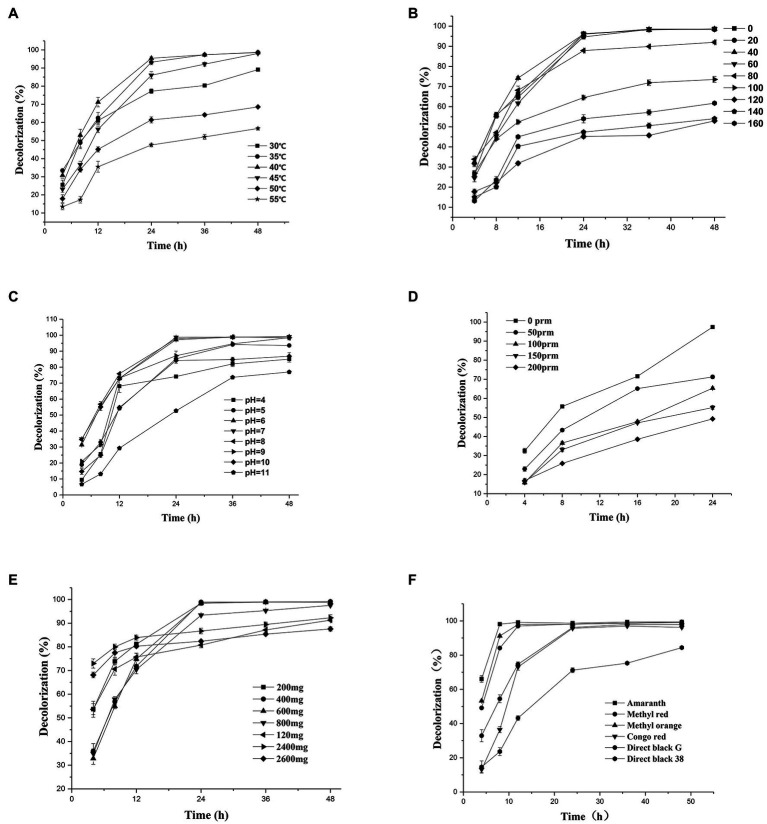
Change in the decolorization efficiency of microflora under different incubation conditions: **(A)** temperature; **(B)** salt concentration; **(C)** initial pH; **(D)** shaker speed; **(E)** Direct Black G (DBG) concentration; and **(F)** different azo dyes. Data points represent the mean of three independent replicates. The standard error of the mean is indicated by error bars.

Temperature plays an important role in bacterial metabolism. Low temperature may lead to insufficient enzyme activity, while high temperature may destroy the active center of the enzyme and inhibit enzyme activity (Jadhav et al., 2008; Chen et al., 2018). Therefore, in this study, temperature was considered as one of the main parameters affecting the decolorization efficiency of the dyes. The halophilic/halotolerant microbial community screened in this study had the ability to degrade azo dyes (i.e., DBG) at 30–55°C (Figure 2A), and the decolorization rate exceeded 98% at 30–40°C. However, with the increase in temperature, the ability of the microflora to decolorize the dyes gradually decreased, but it still maintained 56% degradation ability at 55°C. This may be because the high temperature inhibited the activity of some thermotolerant halophilic bacteria leading to the loss of degradation activity (Jadhav et al., 2008) or the active centers of some enzymes were inactivated at high temperature, resulting in a decrease in the degradation ability. Due to the existence of bacteria with certain heat resistance, such as *Bacillus* and *Enterococcus*, the flora still maintained a certain degradation activity at 55°C. Therefore, the halophilic microflora could adapt to the high temperature used in this study.

#### The Effect of Different Salt Concentrations on the Decolorization Rate

Since osmotic pressure also affects decolorization efficiency, and azo wastewater usually contains a high-salt content, it is important to study the salt tolerance of the microflora. Thus, the effects of different salt contents on the decolorization of azo dyes were tested in this study. The azo dyes at salt concentrations of 0–60 g/l were degraded for 24 h, and the decolorization rate by the bacterial microflora exceeded 95% (Figure 2B). However, the degradation rate of azo dyes at a salt content of 40 g/l NaCl decreased after 12 h compared with that at salt contents 20 g/l NaCl and 60 g/l NaCl. It could be that different concentrations of sodium chloride have different activation effects on different enzymes, leading to the decrease in degradation efficiency and then an increase. As the salt concentration increased further to 100 g/l, the degradation efficiency decreased to 87% after 24 h of treatment and reached > 70% in 48 h. However, with the extension of time, the degradation efficiency increased further, and 61% of the substrate was still degraded at high concentrations of salt (160 g/l). Another reason for the decreased degradation rate at high-salt concentrations could be the reduced solubility of the dye.

#### Effects of Different pH on Decolorization Rate

Because pH plays a key role in the transportation of cell materials across the membrane (Chen et al., 2018), it is generally considered to be one of the main factors affecting the degradation of dyes. A wide range of adaptation to pH was exhibited by the halophilic/halotolerant microflora in this study (Figure 2C). Based on the high pH caused by metal cations in the salt mine and the severe environment in which the bacteria lived, the bacteria in the salt mine environment would be adaptable to a wide range of pH values, which could be more conducive to the survival of the microflora. Figure 2C shows that the microflora had a relatively stable decolorization effect on azo dyes in the pH range of 5–10, and the effective decolorization rate was > 97% in 48 h. However, when the reaction substrate became alkaline, the decolorization rate of the azo dyes by the microflora began to decrease. As the bacteria grew and metabolized, the alkaline environment could be acidified by the various organic acids produced by the bacteria. Therefore, the 48-h degradation rate of azo dyes had little effect in appropriate alkaline conditions. However, when the pH of bacterial culture was > 11, the growth of the microflora was affected, and the degradation rate was significantly lower than that under acidic conditions. Previously, there were reports that the pH of wastewater from printing and dyeing plants was typically 8–9 (Wang et al., 2013). Thus, the azo dyes used by printing and dyeing factories could be degraded by halophilic/halotolerant microflora across a wide range of pH conditions in this study, and the microflora would thus have excellent prospects for industrial application in the future.

#### Effect of Oxygen Content on Decolorization Efficiency

The oxygen content is an important parameter that affects the decolorization rate of extremely halophilic and halotolerant bacteria. In this study, the 24-h decolorization rate of the extremely halophilic/halotolerant flora under static conditions was > 98% (Figure 2D). However, when the rotation speed was 50 rpm, the decolorization rate began to decrease (71.3%), and when it was 200 rpm, the azo dye degradation rate was 49.8%. Because oxygen can compete with the degradation-related enzymes for reaction centers, the decolorization rate decreased with the increase in revolutions, and synchronously, greater shear stress can lead to higher cell destruction rates at high revolutions. These results are consistent with those of Rania Al-Tohamy et al. (2020), who found that when the number of revolutions was 0, the yeast strain SSA-1575 completely decolorized Reactive Black 5 (RB5) within 24 h. However, when the shaker was rotated at 200 rpm, only 13% of RB5 was degraded.

#### Effect of Initial Concentration of Azo Dyes on the Degradation Rate

As shown in Figure 2E, the relationship between the dye decolorization rate and the concentration, as well as the degradation efficiency, was revealed in the experimental results. The initial dye concentration would also affect the degradation efficiency because of the toxicity of azo dyes, particularly for extremely high concentrations of dye. When the concentration of DBG was maintained at 200–600 mg/l, the microflora were effective at degrading DBG and could degrade > 70% after 12 h of incubation (Figure 2E), and the azo dye could be completely degraded after 24 h of incubation. However, as the dye concentration continued to increase, the degradation efficiency began to decrease significantly, and the rate of degradation was reduced with the extension of the culture time. This trend could be caused by the accumulation of intermediate metabolites, the increased toxicity, and the depletion of nutrients and redox media. Simultaneously, the rate of degradation of 2,400 mg/l DBG dye was still > 90% in 18–48 h. However, when the DBG content was 3,600 mg/l, most of the dye precipitated; thus, the consistency of the liquid medium increased, and a large amount of the extracellular polysaccharides were released from the bacteria. The bacteria then clearly displayed signs of extreme toxicity. The experimental results indicate that the decolorization performance was affected by the dye concentration with the increase in toxic intermediate products (Chang et al., 2001; Agrawal et al., 2014). Compared with another halophilic bacterium PHMM that could only adapt to the 10 g/l salt (Mnif et al., 2017), extremely halophilic and halophilic bacteria have the right advantage to degrade higher concentrations of salt-containing azo dye wastewater. Compared with the ability of halotolerant yeast *Pichia occidentalis* G1 (isolated from sea mud), *S. halophilus SSA-1575*, and *Scheffersomyces spartinae* TLHS-SF1 (Tan et al., 2016; Song et al., 2017; Al-Tohamy et al., 2020), as reported by Rania Al-Tohamy et al. (2020) and Tan et al. (2016), the halophilic microflora in this study possessed a stronger ability to tolerate salt and exhibited more potential for industrial applications.

#### Degradation of Dyes With Different Structures by Extreme Halophilic Bacteria

The excellent decolorization of azo dyes with different structures was exhibited in the halophilic/halotolerant microflora in this study. Five azo dyes with different structures at a concentration of 600 mg/l were used to study the decolorization efficiency (Figure 2F). Amaranth, methyl orange, and methyl red could be degraded completely, and 73% of the Congo red was degraded after 12 h of incubation. Congo red is only slightly soluble in water, which could affect its decolorization efficiency. However, all the azo dyes could be decolorized by more than 95% by the microflora in 24 h. Owing to the presence of amino groups (Singh and Arora, 2011), their low molecular weight, and simple structure, Amaranth and methyl red would be conducive to rapid decolorization by microflora (Kurade et al., 2015), and the poor decolorizing ability of black G38 could be the result of differences in its structure. Nevertheless, the decolorization rate of various azo dyes by halophilic microflora could reach > 99%, and the results indicate that the various azo dyes of wastewater could be decolorized.

### Enzymatic Activities

The biodegradation of azo dyes must be controlled by enzymes, so it is imperative to study the enzymes produced by microflora during the biodegradation of azo dyes. This study sought to obtain halophilic and halophilic flora with higher salt tolerance, so we determined the laccase, manganese peroxidase, lignin peroxidase, and NADH–DCIP levels of the microflora at different salt concentrations. The results of the supernatant (extracellular) and bacterial pellet (intracellular) levels before and after DBG decolorization are shown in Table 3. Compared with the control culture, the contents of oxidase, laccase, lignin peroxidase, and manganese peroxidase in the cells increased significantly, indicating that the presence of azo dyes can promote the expression of enzymes related to azo dye degradation (Table 3). In particular, the intracellular laccase and lignin peroxidase were highly induced in the decolorized medium, indicating that the metabolites formed were further oxidized. The role of laccase in the oxidation of azo dyes and its degradation metabolites has been reported before (Oturkar et al., 2011). However, this experiment suggests that the synergistic effect of these enzymes was the cause of DBG decolorization and degradation through the coexistence of these enzymes in this study. The azo bond in the micro-oxygen environment could be reductively cleaved by azo reductase, and peroxidase, lignin peroxidase, and laccase could also be produced by the bacteria, which played a supporting role in azo degradation based on previous studies. In this study, the degradation efficiency of azo by the microflora decreased as the concentration of sodium chloride reached 40 g/l and then increased again at 60 g/l. It is possible that the different enzymes could be activated by different concentrations of sodium chloride, causing different degradation efficiencies. The activities of NADH–DCIP and laccase in 20 and 60 g/l sodium chloride indicate that the levels of expression of enzymes were very different, indicating that the NADH–DCIP enzyme reaction center could be activated and that the enzyme activity was enhanced by higher concentrations of sodium chloride (Samir et al., 2020). There are also reports that NADG–DCIP enzyme activity could be activated, and the degradation of azo dyes is accelerated by high concentrations of sodium chloride (Al-Tohamy et al., 2020).

**Table 3:** Enzyme activities in the supernatant and microbial cells before (control) and after decolorization of 600 mg/l DBG.

Enzyme	Control		20 g/L NaCl		40 g/L NaCl		60 g/L NaCl	
	Int	Ext	Int	Ext	Int	Ext	Int	Ext
Azo reductase^a^	0.9377	0.9683	3.0918±0.00437^**^	7.1304±0.01162^*^	5.7971±0.01162^*^	4.6763±0.00394^**^	2.7440±0.00843	0.5411±0.00422
Lac^b^	0.0036	NA	0.24691±000411	1.04938±0.00254^**^	0.2679±0.00263	0.74074±0.00183^**^	0.53086±0.00263	0.58026±0.00105^*^
Lignin peroxidase^b^	0.0014	NA	3.51852±0.677^*^	0.01624±0.00637^**^	0.51852±0.00496	0.00487±0.00339^*^	1.35802±0.00452	0.00803±0.0039^**^
NADH–DCIP^b^	5.7713	4.7649	28. 5965±0.00401^**^	5.2339±0.00208^**^	33.8596±0.00434^**^	3.83041±0.00568^**^	40.9942±0.00105^**^	7.6318±0.0033^**^
Mn peroxidase^b^	0.0019	NA	0.02292±0.00416	0.15624±0.0229^**^	0.00585±0.00231^*^	0.15735±0.2275^**^	0.01749±00422^**^	0.18409±0.00137^**^

Ext, extracellular; Int, intracellular. Values are the mean of three experiments ± standard error mean (SEM), and significance differences from the control are indicated by *(*p* < 0.01) and **(*p* < 0.001) based on one-way analysis of variance (ANOVA) with a Tukey–Kramer comparison test. ^a^μM of methyl red reduced min^−1^ mg of protein^−1^, ^b^enzyme units min^−1^ mg protein^−1^.

### Degradation Pathway Analysis

**Figure 3:**
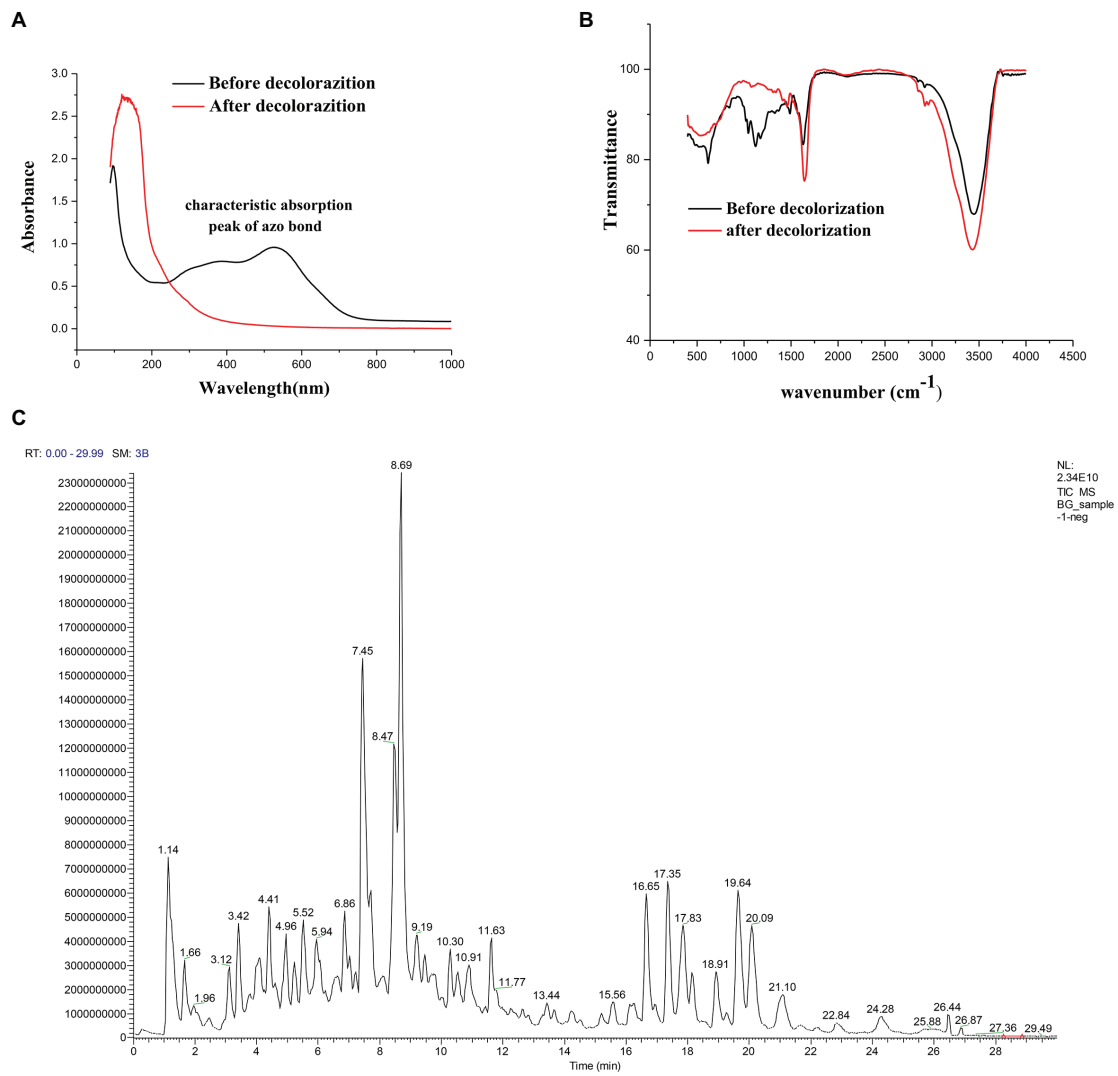
**(A)** UV–Vis spectrum analysis of 600 mg/l Direct Black G (DBG) medium after decolorization of extremely complex halotolerant/halophilic flora. **(B)** Fourier infrared spectroscopy analysis of 600 mg/l DBG medium after decolorization of extremely complex halotolerant/halophilic flora. **(C)** LC–MS analysis of 600 mg/l DBG medium after the decolorization of extremely complex halotolerant/halophilic flora.

To fully investigate the degradation pathways of azo dyes by halophilic/halotolerant microflora, the degradation products of DBG were analyzed by UV–Vis spectroscopy, Fourier infrared spectroscopy, and LC–MS structural identification. The maximum absorption peak was near 645 (visible light region) primarily because of the existence of azo bonds (Figure 3A). After 24 h of biodegradation, the absorption peaks migrated to the ultraviolet and extreme ultraviolet regions. The main reason was that the azo bond in the molecule was degraded, and all the large DBG molecules were degraded to small molecules that contained benzene rings. The absorbance of the peak at 645 nm dropped sharply to 0, indicating that the azo dye had been degraded, and the –N=N– bond had been cleaved (Enayatizamir et al., 2011; Álvarez et al., 2013; Tan et al., 2016; Martorell et al., 2017). Additionally, the disappearance of the high absorption peak in the ultraviolet region indicates that the molecular structure had changed before and after the decolorization, providing evidence of the change in molecular structure.

After degradation by microorganisms, the main staining sites of the DBG molecules were attacked, resulting in the molecular deconstruction. Some of the initial absorption peaks disappeared, while some new absorption peaks were formed (Figure 3B). The absorption peak value of the azo bond is 1,540–1,400 cm^−1^, corresponding with the –N=N– stretched asymmetric azo group. As this area is primarily the color-developing area, the absorption peak of this area disappeared after degradation, which proved that the azo bond was broken. C–C, N–H, O–H, and O–C stretching in the benzene ring were confirmed by the presence of peaks at 1,750–1,620 cm^−1^, 3,697–3,000 cm^−1^, and 1,260–1,080 cm^−1^, respectively, and N–H stretching was also indicated by the absorption peak at 620 cm^−1^. NH_2_ bending was revealed by the absorption peaks at 1,660 and 550 cm^−1^, and C–S and S–O stretching were inferred from the absorption peak at 1,260 cm^−1^. C–H asymmetric stretching in the CH_2_ groups, R–SO_3_ group with =S=O stretching, N–H, alkene, and sulfoxide SO stretching were shown by absorption peaks at 2,970–2,880 cm^−1^, 1,250–1,140 cm^−1^, 1,600–1,390 cm^−1^, 1,070–1,010 cm^−1^, and 1,130–1,070 cm^−1^, respectively. Additionally, the υ3 and υ4 stretching of the SO_4_ groups were located at the peaks of 1,070 cm^−1^ and 660–620 cm^−1^, respectively. The vibrational mode of the [HSO_4_]^−^ anion was indicated by the peak at 850 cm^−1^, and sulfone SO_2_ stretching was inferred from the peak at 1160–1120 cm^−1^. With the extension of degradation time, the CH in the CH_2_ group underwent asymmetric stretching, and =S=O, NH, olefin, and sulfoxide SO stretching occurred in the R–SO_3_ group. Moreover, degradation of the DBG structure was indicated by υ3 and υ4 stretching of the SO_4_ group [HSO_4_]^−^ anion vibration in the SO_2_ stretching and the disappearance of the secondary amine and other structures.

Alternatively, the significant molecular structure changes of the azo dye DBG by the halotolerant and halophilic microflora were indicated by the appearance of some new peaks. To analyze the mechanism of extreme halophilic/halotolerant microflora degrading azo dyes through the degradation of DBG, the composition of the degradation products of DBG was identified and detected by mass spectrometry (Figure 3C). Owing to the presence of *Bacillus* in the mixed microflora, there were many metabolites and complex components, so there were many miscellaneous peaks in the LC–MS results. Several possible metabolites were determined based on the corresponding mass spectra of the extracted products after the degradation of DBG (Table 4).

**Table 4:** Characteristics of the main azo dyes used in this study.

Serial number	Time (min)	Molecular weight	Precursor ions (m/z-structure)	Molecular formula	Chemical structures	Chemical name
1	3.97	189	190	C_10_H_11_N_3_O	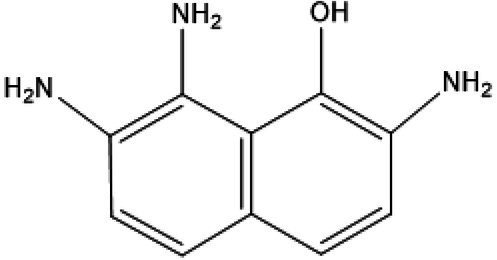	2,7,8-Triaminonaphthalen-l-ol
2	2.73	176	179	C_10_H_18_O_3_	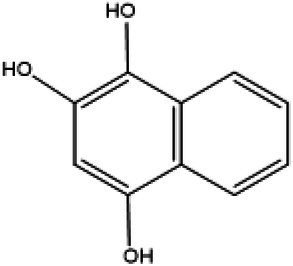	Naphthalene-1,2,4-triol
3	1.28	110	109	C_6_H_6_O_2_	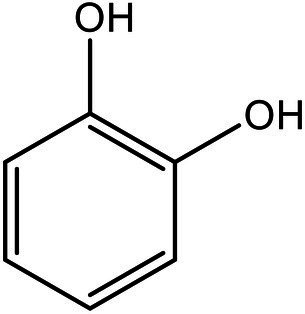	Catechol
4	3.58	108	109	C_6_H_9_N_2_	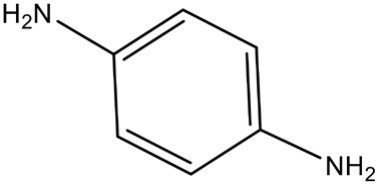	*p*-Phenylenediamine
5	4.08	166	165	C_8_H_6_O_4_	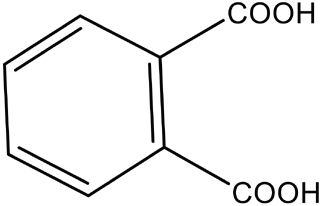	Phthalic acid
6	3.12	132	131	C_5_H_8_O_4_	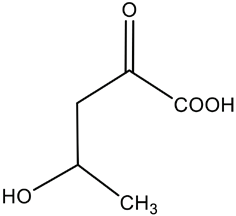	4-Hyydroxy-2-oxopentanoic acid
7	0.39	94	95	C_7_H_6_O_4_	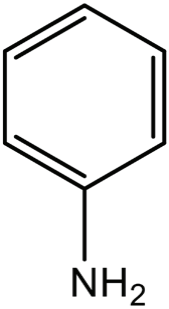	Aniline

**Figure 4:**
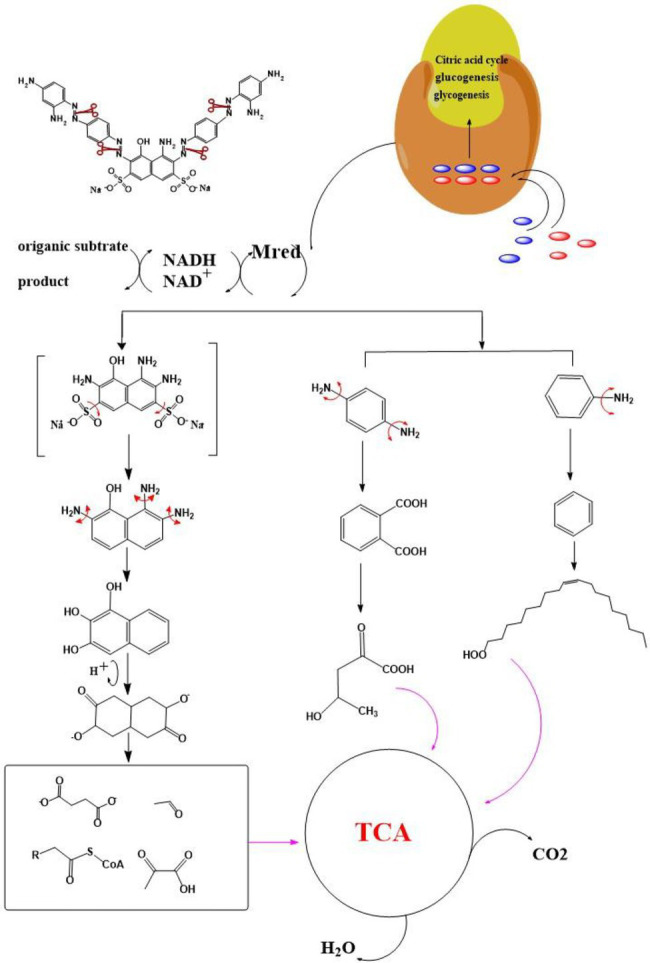
Proposed pathway for the degradation of DBG by halophilic/halotolerant bacteria.

After the DBG decolorization reaction, six possible metabolites were identified based on the corresponding mass spectra and m/z values of the extraction solutions. Mass spectral retention times obtained after DBG degradation revealed possible metabolites. The degradation pathways follow either symmetric or asymmetric degradation (Jain et al., 2012). Based on mass spectrometry analysis, a possible pathway for DBG degradation was proposed (Figure 4). The biodegradation process is initiated by the cleavage of the azo bond (Franciscon et al., 2015). The reducing force generated by the carbon source and the nitrogen source catalyzes the cleavage of the azo bond of DBG by azo reductase, resulting in the fading of the dye color, which is usually considered the first step in the degradation of azo dyes (Song et al., 2018). After the dye is degraded, owing to the instability of the sulfonic acid group, it may quickly degrade into other by-products (Qu et al., 2012; Tan et al., 2016; Song et al., 2018), and the decolorization reaction product 2,7,8-triaminonaphthalene-1-ol (product 1) may be deaminated to naphthalene-1,2,4-triol (product 2), which may be converted to catechol (product 5), which is oxidatively cleaved *via* the cis-mucic acid pathway into aliphatic metabolites (Ferraroni et al., 2004; Oturkar et al., 2011), followed by further oxidative conversion to smaller compounds. The initial degradation products of DBG by the flora also include *p*-phenylenediamine (product 4), and the other metabolites are phthalic acid (product 5) and 4-hydroxy-2-oxopentanoic acid (product 6). The loss of amino compounds (–NH_2_) from DBG is caused by deamination (Patel et al., 2013). Aromatic amines produced during dye decolorization can be further degraded into smaller compounds and may eventually be mineralized (Oturkar et al., 2011; El Bouraie and El Din, 2016; Martorell et al., 2017; Zhuang et al., 2020). The presence of these products indicates cleavage of the azo bond of the dye DBG. On this basis, a possible pathway for the degradation of DBG can be proposed. After DBG is degraded by azo reductase, three intermediates are generated as: 2,7,8-triaminonaphthalen-1-ol (molecular weight 189, m/z 190), *p*-phenylenediamine (molecular weight 108, m/z 109), and aniline (molecular weight 93, m/z 94), which are further converted to low molecular weight compounds *via* oxidation by laccase, manganese peroxidase, and lignin peroxidase. As the degradation process continues, 2,7,8-triaminonaphthalen-1-ol is further degraded to phthalic acid (molecular weight 166, m/z 167). A similar response was also reported by Xu et al. (2009). At the same time, the intermediate of *p*-phenylenediamine is deaminated to generate aniline, which is then subjected to a ring-opening reaction to generate 4-hydroxy-2-oxopentanoic acid (molecular weight 132, m/z 131; Zhang, 2011). From the findings obtained by UV–Vis, FTIR, and mass spectrometry analysis, it can be concluded that the RB5 decolorization of strain SSA-1575 occurs through cleavage of the azo bond, resulting in the formation of colorless aromatic amines, but they can be further degraded into smaller compounds, which may eventually be mineralized as H_2_O and CO_2_ (Schweigert et al., 2001; Figure 4).

### Toxicity Studies

After DBG was decolorized, DBG was subjected to ecotoxicological evaluation to evaluate the safety of its DBG metabolites. Acute toxicity assessment was performed to evaluate the acute toxicity of DBG decolorization intermediates from halophilic flora at high-salt concentrations.

**Figure 5:**
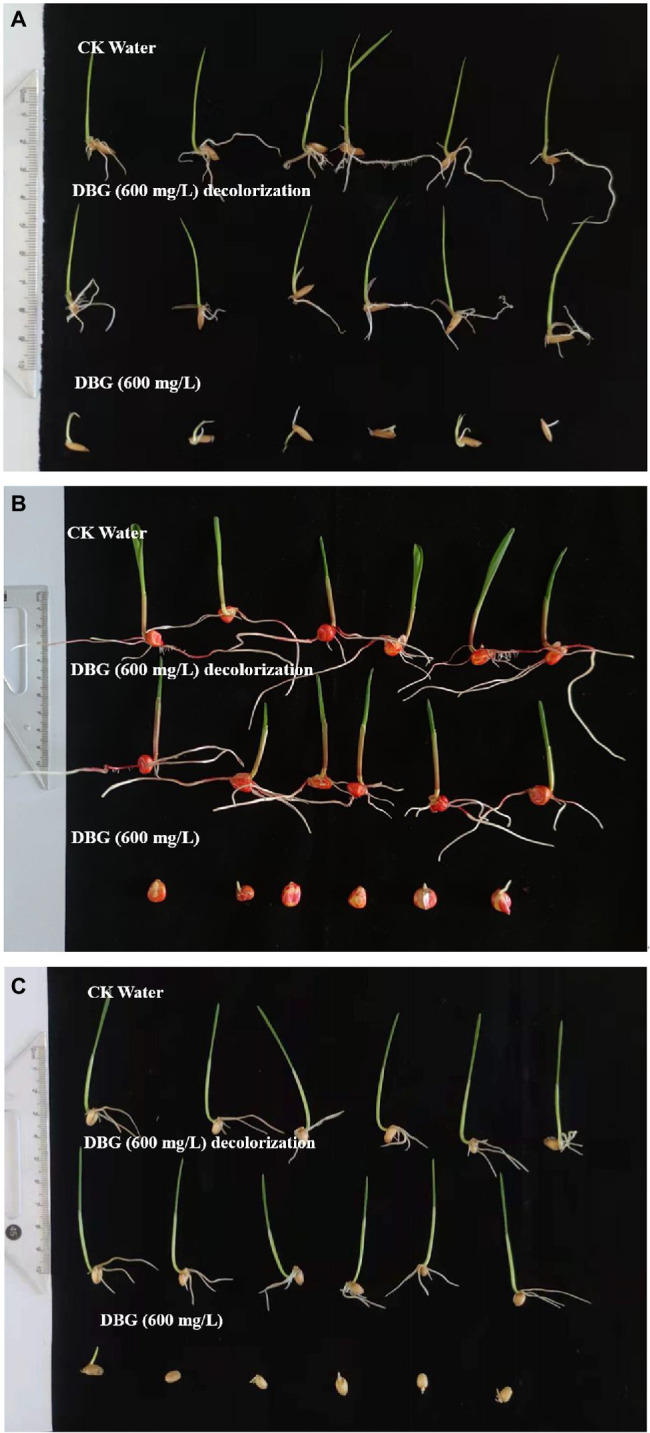
Representative images from the biological toxicity test with **(A)** rice, **(B)** corn, and **(C)** wheat.

Three agriculturally important types of seeds were used to test the biological toxicity of DBG metabolites produced by the microflora, and the results are shown in Figure 5. The 100-seed germination rate of the DBG-treated rice, corn, and wheat seeds was significantly lower than that of the distilled water and the decolorized treatment groups (Table 5). The toxicity test showed that when the seeds were treated with 600 mg/l DBG, the germination rates of rice, corn, and wheat only reached 56.3, 47.4, and 42.5%, respectively. Simultaneously, shorter shoot lengths (0.41, 0, and 0.21 cm, respectively) and root lengths (0.22, 0.35, and 0.37 cm, respectively) were also observed. However, the germination rate of seeds in the supernatant of DBG degraded by the microbial flora could reach more than 100%, and the supernatant was desalinated by dialysis. Simultaneously, it was observed that the seeds had a longer shoot length after germination (4.35, 6.04, and 4.68 cm, respectively) and root length (5.58, 7.37, and 2.51 cm, respectively), and the seeds grew well. The germination rate of seeds irrigated with distilled water in the control group was 100%, and the shoot length (4.72, 6.18, and 4.73 cm, respectively) and root length (5.67, 7.22, and 2.53 cm, respectively) did not differ significantly from the treatment group. Therefore, the toxic dye DBG could be degraded to metabolites of low toxicity by the microflora in this study based the results of the phytotoxicity analytical experiments. Thus, extreme halophilic/halotolerant flora may serve as an effective treatment for DBG contamination that can be safely implemented in bioremediation processes, especially for high-salt azo dye wastewater.

**Table 5:** Phytotoxicity of dye DBG (600 mg/l) and its degradation products extracted after degradation (48 h).

Sample	Rice (cm)			Corn (cm)			Wheat (cm)		
	Germination (%)	Shoot	Root	Germination (%)	Shoot	Root	Germination (%)	Shoot	Root
Distilled water	100	5.16 ± 0.38	5.76 ± 0.31	100	6.78 ± 0.56±	7.63 ± 0.33	100	5.01 ± 0.48	2.77 ± 21
DBG	56.3	0.44 ± 0.27^*^	0.18 ± 0.01^*^	47.4	0.00	0.38 ± 0.24^*^	42.5	0.19 ± 0.35^*^	0.33 ± 0.15^*^
Degradation products	100	4.56 ± 029^%^	6.33 ± 0.39^%^	100	6.40 ± 0.45^%^	7.37 ± 0.32^%^	100	4.77 ± 0.37	2.64 ± 0.1.5^%^

Values are the mean of three experiments ± standard error mean (SEM); data were analyzed by one-way analysis of variance (ANOVA) with a Tukey–Kramer multiple comparison test using the mean values of germinated seeds of three experiments. Seeds germinated in DBG differed significantly from the seeds germinated in distilled water at *p < 0.01, and the seeds germinated in degradation products differed significantly from the seeds germinated in DBG at ^%^*p* < 0.5.

## Conclusion

Halophilic/halotolerant microflora that could efficiently degrade and detoxify azo dyes was successfully developed, and this study shows that they had excellent effects on a variety of azo dyes under the synergistic action of laccase, manganese peroxidase, DIIP–NADH reductase, lignin peroxidase, and azo reductase. Moreover, based on the identified intermediates, a new mechanism to degrade DBG through this halophilic/halotolerant microflora was proposed. The LC–ESI–MS phytotoxicity study further showed that DBG could be degraded into less toxic metabolites through the action of these microorganisms. This complex halophilic/halotolerant flora has practical prospects to treat azo dye wastewater.

## Data Availability Statement

The data presented in the study are deposited in the NCBI Sequence Read Archive (SRA) database, accession number: SAMN23288608- SAMN23288610.

## Author Contributions

HQ and FS contributed equally to this work. All authors contributed to the article and approved the submitted version.

## Funding

This work was supported by the Guangdong Basic and Applied Basic Research Foundation (2019A1515011696), the Guangdong Special Project on Key Fields of Colleges and Universities (Rural Revitalization, 2020ZDZX1020), and the National Natural Science Foundation of China (31760438).

## Conflict of Interest

The authors declare that the research was conducted in the absence of any commercial or financial relationships that could be construed as a potential conflict of interest.

## Publisher’s Note

All claims expressed in this article are solely those of the authors and do not necessarily represent those of their affiliated organizations, or those of the publisher, the editors and the reviewers. Any product that may be evaluated in this article, or claim that may be made by its manufacturer, is not guaranteed or endorsed by the publisher.
